# COVID-19 mRNA Vaccine Tolerance and Immunogenicity in Hematopoietic Stem Cell Transplantation Recipients Aged 5–11 Years Old–Non-Randomized Clinical Trial

**DOI:** 10.3390/vaccines11010195

**Published:** 2023-01-16

**Authors:** Agnieszka Matkowska-Kocjan, Joanna Owoc-Lempach, Kamila Ludwikowska, Filip Szenborn, Natalia Moskwa, Katarzyna Kurek, Krzysztof Kałwak, Leszek Szenborn, Marek Ussowicz

**Affiliations:** 1 Department and Clinic of Pediatric Infectious Diseases, Wroclaw Medical University, 50-368 Wrocław, Poland; 2 Department and Clinic of Paediatric Oncology, Haematology and Bone Marrow Transplantation, Wroclaw Medical University, 50-556 Wrocław, Poland; 3 Faculty of Electronics, Wroclaw University of Science and Technology, 50-370 Wrocław, Poland

**Keywords:** COVID-19, vaccinations, HSCT, children

## Abstract

The SARS-CoV-2 pandemic had a devastating impact on the world’s population in the years 2020–2022. The rapid development of vaccines enabled a reduction in the mortality and morbidity of COVID-19, but there are limited data about their effects on immunocompromised children. The aim of this prospective study was to evaluate the safety and efficacy of the mRNA BNT162b2 (Pfizer/Biontech) vaccine in allogeneic hematopoietic stem cell transplantation (allo-HSCT) recipients. Material and methods: Two cohorts of 34 children after allo-HSCT and 35 healthy children aged 5–11 years were vaccinated with two doses of the mRNA BNT162b2 (10 µg) vaccine. All children were evaluated for adverse effects with electronic surveys and the immunogenicity of the vaccine was assessed with anti-SARS-CoV-2 IgG titer measurements. Results: All reported adverse events (AEs) were classified as mild. The most common AE was pain at the injection site. All the other AEs (both local and systemic) were rarely reported (<15% patients). Both groups showed a similar response in anti-SARS-CoV-2 IgG production. Patients after allo-HSCT that were undergoing immunosuppressive treatment presented a poorer immunological response than patients off of treatment. Time since HSCT, patient age, lymphocyte count, and total IgG concentration did not correlate with initial/post-vaccination anti-SARS-CoV-2 IgG titers. Most patients who were eligible for a third dose of the vaccine had an excellent humoral response observed after two vaccine doses. Conclusions: The COVID-19 mRNA BNT162b2 vaccine is very well tolerated and highly immunogenic in 5–11-year-old children after HSCT. Children >2 years of age after HSCT who did not receive immunosuppressive treatment presented excellent antibody production after two doses of the vaccine, but children on immunosuppression may require a more intense vaccination schedule.

## 1. Introduction

The SARS-CoV-2 pandemic had a devastating impact on the world’s population in the years 2020–2022. The greatest morbidity and mortality from COVID-19 has been seen among older adults and patients suffering from chronic diseases, such as conditions connected with immunosuppression which have appeared to be a significant risk factor for severe COVID-19 [1,2,3]. Therefore, non-immunocompetent patients were considered as a priority group for rapid anti-SARS-CoV-2 immunization after the start of the COVID-19 vaccination campaign in December 2020. In most countries, moderately and severely immunocompromised adult patients received access to COVID-19 mRNA vaccines in early spring 2021 regardless of their age, and this was the most important factor when deciding early access for the general population. Soon after the start of vaccination, many immunosuppressed patients presented lower immune response to the mRNA COVID-19 vaccines than the general population, thus requiring additional vaccine doses in the primary vaccination schedule [4]. In August/September 2021, most countries recommended an additional dose of an mRNA COVID-19 vaccine for immunosuppressed patients, with this group including allogeneic hematopoietic stem cell transplantation (allo-HSCT) recipients [5]. A third vaccine dose was recommended after a minimum of 28 days from the second dose of the mRNA vaccine. The criteria for the three-dose primary vaccination schedule for allo-HSCT recipients were defined as follows: a less than 2-year interval from the allo-HSCT and/or ongoing immunosuppressive treatment. This recommendation was based on the observations of adult and teenage (>12 years old) post-transplant patients, who showed suboptimal humoral response. At the end of 2021, a COVID-19 mRNA vaccine (BNT162b2) was registered in the European Union for children in the age group of 5–11 years old [6]. All children at this age were recommended to get vaccinated, but special considerations applied to children with comorbidities, allo-HSCT recipients among them. The guidelines for the extended three-dose primary vaccination schedule were extrapolated from experience with the older population [7]. At the time of licensing, there were no published data regarding the tolerance, immunogenicity, and optimal vaccination schedule of the vaccine in younger pediatric HSCT patients. Therefore, we decided to study the feasibility of the mRNA BNT162b2 (Comirnaty, Pfizer/Biontech, Mainz, Germany) vaccine in 5–11-year-old children after allo-HSCT.

## 2. Materials and Methods

Thirty-nine children aged between 5 and 11 years old who had undergone an allo-HSCT (allo-HSCT group) and thirty-five healthy children (CONTROL group) of similar age were included in the prospective observational cohort study. Patients from the allo-HSCT group were recruited from 160 children aged 5–11 years old who had undergone an allo-HSCT and continued follow-up care in the largest pediatric bone marrow transplantation centre in Poland. The parents/legal guardians of those children were contacted by experienced infectious diseases specialists and/or pediatric oncologists and encouraged to get the child vaccinated against COVID-19, and if the parent was willing to get the child vaccinated, then participation in the study was offered. The children from the HSCT group and the control group were vaccinated with 2 doses of the mRNA Comirnaty (10 µg, Pfizer/Biontech) vaccine at 3-week intervals. The 2-dose vaccination schedule was performed between 18 December 2021 (the first patient vaccinated with the first dose) and 29 January 2022 (the last patients vaccinated with the second dose). The patients who were infected with SARS-CoV-2 between the first and the second vaccination were withdrawn from further observation. A total of 15 patients from the allo-HSCT group who met the criteria for the additional vaccine dose and showed poorer response after the second vaccine dose were administered the third dose after 4–6 weeks from the second dose (between 28 February 2022 and 28 March 2022). All study participants were evaluated for the prevalence and severity of self-reported adverse events (AEs) and the immunogenicity of the vaccine.

### 2.1. Recording of Adverse Events

All patients were asked to fill in an electronic survey every day for the first 7 days after each dose of the vaccine, once per week between the vaccine doses, and for 1 week after the second dose. The reminders about the survey were sent daily in the evening (through mobile phones or e-mails). Each reminder contained a personalized link to the online survey, which was part of a dedicated web application designed for collecting data. The patients were asked about the presence of systemic symptoms such as fatigue, headache, chills, new or worsening muscle or joint pain, vomiting/nausea, low-grade fever, and fever, as well as the presence of local reactions such as pain, redness, swelling, induration or pruritus at the injection site, and the presence of axillary lymphadenopathy. When an adverse event was reported, the patients were asked to define the severity of the symptom. The reported AEs were classified and graded according to the Common Terminology Criteria for Adverse Events (CTCAE) [8].

### 2.2. Immunological Assays

**Figure 1:**
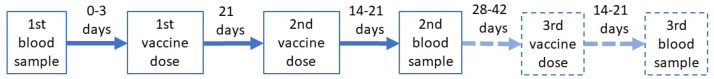
The vaccination scheme.

During the last pre-vaccination visit, a blood sample was collected for lymphocyte subpopulation assessment. The peripheral blood sample was immunophenotyped by multicolor flow cytometry and the number of T, B lymphocyte, and NK cells was determined using the BD MultitestTM 6-Color TBNK reagent (cat no. 337166, BD Biosciences, San Jose, CA, USA) with fluorochrome-conjugated mouse monoclonal antibodies CD3-FITC (clone SK7), CD16-PE (clone B73.1), CD56-PE (clone NCAM16.2), CD45-PerCP-Cy5.5 (clone 2D1), CD4-PE-Cy7 (clone SK3), CD19-APC (clone SJ25C1), and CD8-APC-Cy7 (clone SK1). Lymphocyte evaluation was performed using a FACSCanto cytofluorimeter (BD Biosciences, San Jose, CA, USA). The serum samples for anti-SARS-CoV-2 IgG antibody analysis were collected 3 to 0 days before the first vaccine dose in all the patients and 14–21 days after the second (and in eligible patients after the third) dose of the vaccine. Children vaccinated with the third dose were sampled again 14–21 days after the third dose. The scheme is presented in Figure 1.

Anti-SARS-CoV-2 antibody levels were measured with the chemiluminescent microparticle immunoassay (CMIA) “Alinity I” from Abbott Diagnostics (Abbott Park, IL, USA), which is a two-step qualitative and semi-quantitative assay designed to detect IgG antibodies against the receptor binding domain of the S1 subunit of the spike protein of SARS-CoV-2 in serum or plasma. Positive concentration values were defined as >7 BAU/mL. The upper detection limit of the test was limited at 5680 BAU/mL, thus for study purposes high anti-SARS-CoV-2 values were rounded to 5680 BAU/mL. From each patient, basic demographic and clinical data were also collected, including sex, age, diagnosis, time from allo-HSCT, the presence of chronic graft-versus-host disease (cGvHD), immunosuppression use, and history of COVID-19 diagnosis. The patient characteristics are presented in Table 1.

### 2.3. Statistical Methods

Differences in categorical data were studied using the two-tailed Fisher’s exact test. Comparisons of the pre- and post-vaccination anti-SARS-CoV-2 antibody concentrations between different groups were performed using the Mann–Whitney test. The relationship between anti-SARS-CoV2 antibody concentration and time since HSCT or age were studied with linear regression analysis. Statistical analysis and data presentation were performed with the computer software GraphPad Prism 6.07 (GraphPad Software, La Jolla, CA, USA) and Statistica 13.0 (Statsoft/Dell, Palo Alto, CA, USA). A *p* value less than 0.05 was considered significant.

## 3. Results

**Figure 2:**
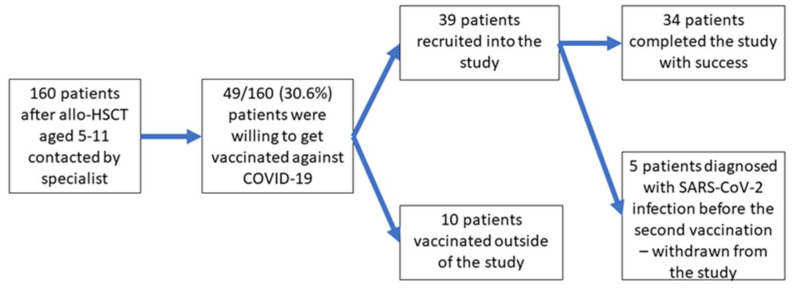
Flowchart of the study.

In total, 49 of the 160 invited parents declared their willingness to vaccinate their child (vaccine acceptance of 36.9%). The rest were hesitant about the vaccination despite a detailed explanation of the COVID-19 vaccination’s benefits. Many of the parents contacted were afraid of the “new” vaccine, and even declared strong and general refusal to allow their child to be vaccinated. Finally, thirty-nine children after allo-HSCT were recruited into the study, and the remaining ten patients decided to get vaccinated outside of the study. Five of them were infected with SARS-CoV-2 between the first and second dose of the vaccine and were withdrawn from further observation; therefore, thirty-four children completed all study procedures and were analyzed (Figure 2).

All five cases of COVID-19 reported in the study between the first and second dose were mild. No SARS-CoV-2 infections were diagnosed after the second vaccine dose during the 2-month follow-up. In the study group, 23/34 children presented positive anti-SARS-CoV-2 IgG antibodies before vaccination, but only 8/23 children suffered from proven SARS-CoV-2 infection before vaccination; all these cases were described by the parents as mild. The details of anti-SARS-CoV-2 antibody concentration and the response to the vaccine are presented in Table 2.

**Figure 3:**
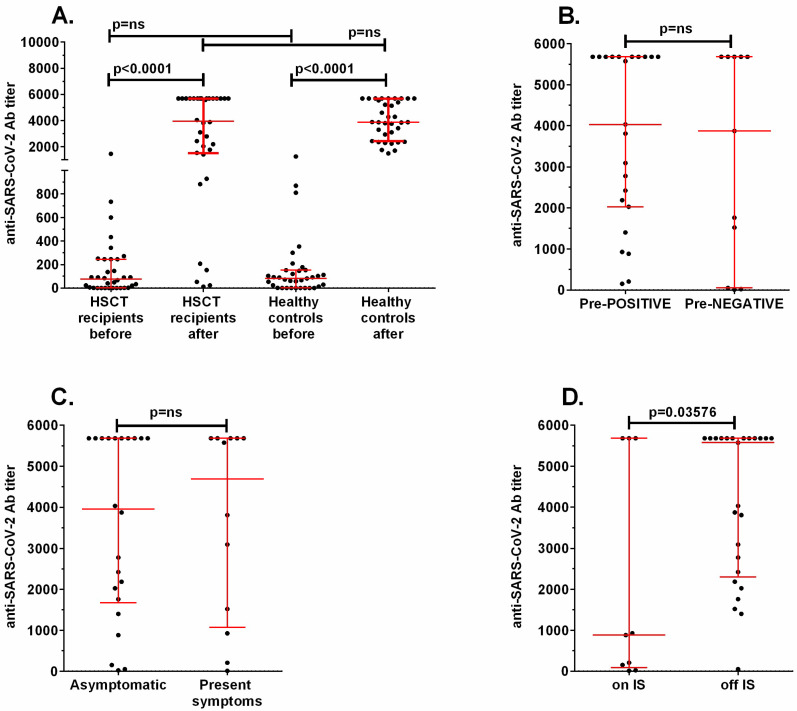
Comparison of the concentration of anti-SARS-CoV-2 specific antibodies in (**A**) HSCT recipients and healthy controls before and after vaccination, (**B**) patients positive before the first dose (Pre-POSITIVE) versus negative (Pre-NEGATIVE), (**C**) patients showing vaccination-associated symptoms and those who were asymptomatic, and (**D**) patients on immunosuppressive treatment (on IS) and those off of treatment (off IS). ns: non-significant.

The comparison of anti-SARS-CoV-2 IgG antibody concentrations before and after vaccination did not show differences between allo-HSCT recipients and healthy controls (Figure 3A). In the allo-HSCT group, the median concentrations were 75.4 BAU/mL (range 0.1–1448) before and 3596 BAU/mL (range 11.5–5680) after vaccination, and in the control group concentrations were 81.03 BAU/mL (range 0.03–1247) and 3868 BAU/mL (range 1481–5680), respectively. The allo-HSCT recipients who were positive for anti-SARS-CoV-2 IgG before vaccination (>7 BAU/mL) did not show a different response after two doses of the vaccine in comparison to initially negative patients (median titer 4035 vs. 3876, *p* = ns, Figure 3B). Patients displaying vaccination-associated AEs showed a similar humoral response to those who were asymptomatic (median 4692 vs. 3956 BAU/mL, *p* = ns, Figure 3C). A statistically significant difference in anti-SARS-CoV-2 IgG response was observed only between patients on immunosuppressive treatment (median 883, range 11.5–5680 BAU/mL) and those off of treatment (median 5574, range 53–5680 BAU/mL) (*p* = 0.0358, Figure 3D).

**Figure 4:**
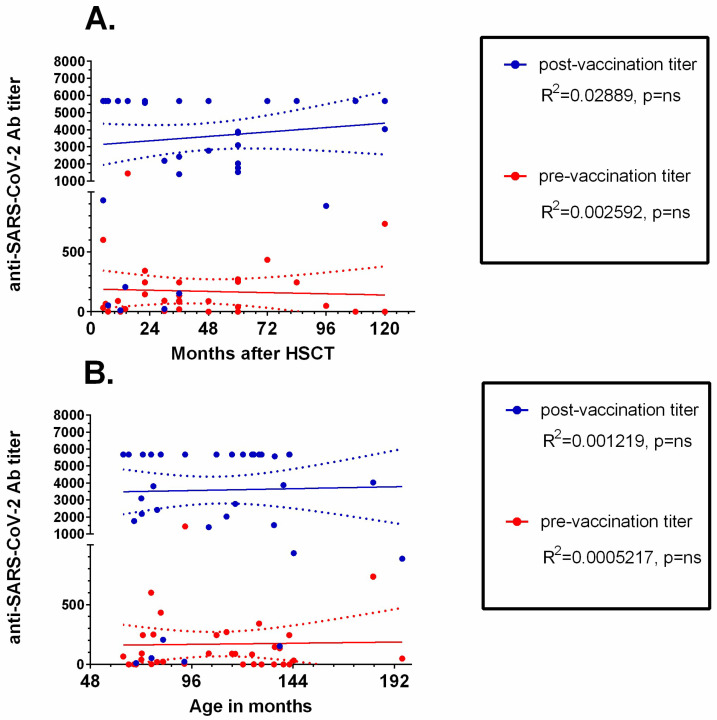
Linear regression analysis of anti-SARS-CoV-2 IgG concentration and (**A**) time since HSCT and (**B**) age of the patient. Trend lines are shown as continuous lines, and dotted lines represent 95% confidence intervals. ns: non-significant.

Both time since HSCT (Figure 4A) and patients’ age (Figure 4B) did not correlate with either initial or post-vaccination anti-SARS-CoV-2 IgG concentrations.

Analysis of pre-vaccination lymphocyte counts and total IgG concentration did not reveal any correlations with final anti-SARS-CoV-2 IgG titers (Table 3).

In the allo-HSCT group, 15 patients fulfilled the official criteria for the third dose of the vaccine. The details concerning this group are presented in Table 4.

In 8 of 15 patients eligible for the third vaccine dose, the anti-SARS-CoV-2 IgG concentrations exceeded the median result observed in the control group.

All reported AEs were classified as mild (grade 1 CTCAE), and the details are presented in Table 5. There was no difference in the incidence of AEs between the allo-HSCT and control groups. 

## 4. Discussion

To the authors’ knowledge, this is one of the first studies concerning SARS-CoV-2 vaccination in children aged 5–11 after allo-HSCT. Many papers concerning the older allo-HSCT population have been published, since vaccines for >18-year-old patients and then >12-year-old adolescents were available earlier (in Poland in March 2021 and June 2021, respectively) [9,10,11,12,13,14,15] than they were for 5–11-year-old children. It should be noted that the published data concerning the immunogenicity and efficacy of primary vaccination schedules in adult allo-HSCT patients cannot be reliably compared to the experience of the pediatric population, since the vaccinations of adults and 5–11-year-old children were separated by 9 months, during which two massive waves of SARS-CoV-2 infection were observed that had a huge impact on the initial anti-SARS-CoV-2 IgG status of the patients. Additionally, the dose of the Comirnaty vaccine (30 µg) for >12-year-old patients is three times bigger than the dose for 5–11-year-old children (10 µg).

### 4.1. Immunogenicity of the Two-Dose Vaccine Schedule

Since the start of the COVID-19 pandemic, there has been a strong desire to discover the immune biomarker of protection against SARS-CoV-2 [16,17]. Although humoral immunity seems to correlate with resistance against COVID-19, after 2 years of the pandemic and more than 1.5 years of COVID-19 vaccine availability, the protective antibody concentration threshold has not been confirmed [18,19]. Bearing in mind that new variants of SARS-CoV-2 are still emerging and that the composition of the vaccine has not been changed since the start of the epidemic (as of May 2022), in real-life experience an unquestionably protective antibody concentration cannot be determined. The published data suggest that the humoral immunity threshold for the pre-Omicron variants may range from 154 to 171 BAU/mL [16,20] and perhaps even up to 1700 BAU/mL [21]. No studies have yet reported an Omicron-specific correlate of protection [18]. Considering the published data, in our study all healthy children presented excellent immunologic response after completion of the vaccination schedule (at least 1481 BAU/mL). However, in the allo-HSCT group, antibody concentrations lower than 1000 BAU/mL in 7/34 children might have had a suboptimal protective effect. In the absence of a better immunity correlate of COVID-19 control, anti-SARS-CoV-2 IgG concentration may be implemented in decision making for children following allo-HSCT. Although we cannot exclude the possibility that patients with lower immunologic response were sufficiently protected against SARS-CoV-2 infection, the level of anti-SARS-CoV-2 IgG after vaccination may be helpful when deciding on the implementation of individual preventive measures (e.g., masks, distancing/avoiding crowded places, the prophylactic use of monoclonal antibodies such as tixagevimab and cilgavimab) [22]. In our study, the only factor associated with impaired immune response to the vaccine was the administration of immunosuppressive treatment. Even in patients receiving immunosuppression, five out of nine patients responded very well, and this fact can support the decision to offer vaccinations to patients with profound immune impairment. The immune competence of allo-HSCT recipients did not correlate with basic lymphocyte subpopulation counts or total IgG concentration (which were routinely analyzed in the study), but the limited number of allo-HSCT recipients might be responsible for this relationship not being detected. According to studies in adults, peripheral counts of T helper and B cells correlated with humoral response to the mRNA BNT162b2 vaccine in actively treated cancer patients, kidney transplant recipients, and hemodialysis patients [23,24].

This study did not include an analysis of cellular immune response to the vaccine, though there is evidence that cellular immunity plays a role both in COVID-19 prevention and inflammatory response leading to tissue injury [25]. The role of humoral response in protection against COVID-19 in the allo-HSCT recipients is not determined; however, post-vaccination anti-SARS-CoV-2 IgG titers might offer an easily measurable biomarker of immune response. In a study by Oyaert et al., a correlation between anti-SARS-CoV-2 antibody titers and cellular response was reported both in healthy controls and immunocompromised patients [26]. In addition, studies among immunocompromised patients show that even though the serological response to SARS-CoV-2 vaccine is severely impaired in patients treated with B cell-targeting therapy, the majority of these patients develop sufficient cell-mediated immunity [27].

### 4.2. Indications for the Third Vaccine Dose in Allo-HSCT Patients

According to the recommendations, the primary three-dose vaccination schedule is indicated for allo-HSCT patients who have received a stem cell transplant within the last 2 years or those who are still receiving immunosuppressive treatment (e.g., corticosteroids, cyclosporine, mofetil mycophenolate). These recommendations were based on the reported lower immunogenicity of the mRNA COVID-19 vaccines in immunosuppressed patients [28,29,30]. Our results show that children more than 2 years removed from allo-HSCT and those off of immunosuppressive treatment showed excellent humoral response after two doses of the vaccine and were indistinguishable from the healthy controls. It may be stated that the criteria for the third dose have very good negative predictive value in this age group. Even in children fulfilling the criteria for the third dose (15 patients), many showed sufficient vaccine response after two vaccine doses. All eight children with good humoral response initially presented with a positive anti-SARS-CoV-2 IgG result, which proves their previous contact with SARS-CoV-2 and might explain why they showed a secondary immune response to the antigen encoded by the genetic material in the vaccine. The remaining seven patients were vaccinated with the third dose and remained alive and well after completion of the study. Based on our observations, it is worth considering that even immunocompromised patients who are seropositive for SARS-CoV-2 before vaccination might be able to achieve sufficient protection after two doses of the vaccine. This approach is more likely to be accepted by parents showing vaccination hesitancy.

### 4.3. Adverse Events after Vaccination

Our data show that tolerance toward the BNT162b2 vaccine in children aged 5–11 was very good both in healthy children and children following allo-HSCT, which is in agreement with the results of clinical trials in this age group [31,32]. Comparing our experience with studies in adult patients vaccinated with the 30 µg BNT162b2 vaccine, it becomes clear that children tolerate vaccination even better than adults [14,33]. Systemic reactions such as headache or fatigue are seen in more than 40% of patients in the adult population after the first dose and more than 50% of patients after the second dose of the vaccine [34], whereas no child complained of fatigue and less than 15% complained of headaches in our study. The most commonly reported adverse effect in our study was mild, short-lasting pain at the injection site, which is typical for vaccinations. A documented good safety profile may be valuable for parents who are hesitating about getting their child vaccinated due to the fear of unfounded complications.

### 4.4. SARS-CoV-2 Infection after Inclusion in the Study

**Figure 5:**
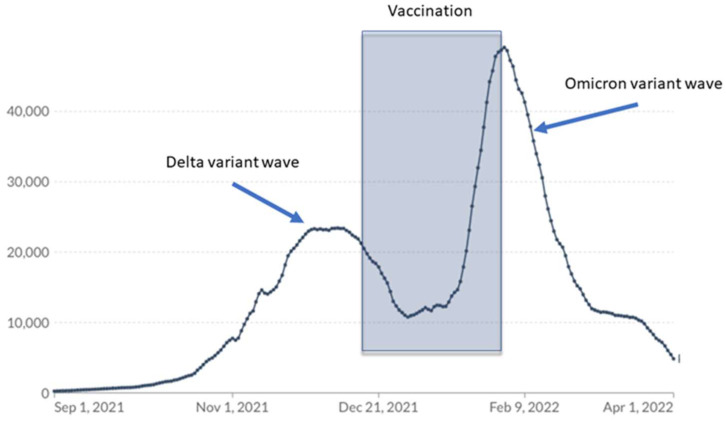
The time patients in the study were vaccinated versus COVID-19 cases reported in Poland.

There were five COVID-19 cases that were diagnosed after the first vaccine dose in patients following allo-HSCT (these patients were further withdrawn from the study); however, there were no reported cases among patients who finished the two-dose vaccination schedule in the 2 months that followed. Our patients were vaccinated in the period between the Delta wave descending and the massive SARS-CoV-2 Omicron variant wave (Figure 5).

The fact that no symptomatic COVID-19 cases were reported among two-dose recipients in February and March 2022, when contact with SARS-CoV-2 was highly probable, is in agreement with other authors’ observations regarding the effectiveness of BNT162b2 against Omicron-variant infections in the first weeks after the completion of the vaccination schedules [35].

### 4.5. Parents’ Willingness to Vaccinate Their Children

Although initially we did not intend to study parental attitudes to the idea of COVID-19 vaccination, while observing the study progress we decided to include this issue. Whilst recruiting patients to our study, we faced unexpectedly low interest in COVID-19 vaccination among the parents of allo-HSCT recipients. In a systematic review and meta-analysis conducted to estimate parents’ and guardians’ willingness to vaccinate their children against COVID-19, F. Chan et al. found that the estimated worldwide vaccination acceptance rate was 61.40% (ranging from 21.6% to 91.4% depending on geographical and cultural factors) [36]. Italian researchers who studied vaccine willingness and hesitancy among parents of children aged 5–11 with chronic diseases (among them hematologic and oncological conditions) showed an even lower acceptance rate. During a study performed in a similar time period as ours (December 2021/January 2022, just after the introduction of COVID-19 vaccines in 5–11-year-old children) they found that only 38.8% of parents were willing to vaccinate their children against COVID-19, which is close to our result [37]. The phenomenon of COVID-19 vaccine hesitancy among allo-HSCT patients was reported earlier by M. Skeens et al. [38]. These observations support the need for educational programs concerning COVID-19 vaccination for children with chronic conditions.

### 4.6. Limitations of the Study

Our study was conducted in a small population of allo-HSCT recipients. However, recruiting children between the ages of 5 and 11 was a challenge due to COVID-19 vaccine hesitancy. We also wanted to enroll patients in the study in the shortest possible time period due to the looming wave of the highly infectious Omicron variant (Figure 5).

The next limitation of the study is that we did not measure cellular immune response to the COVID-19 vaccines and only focused on humoral response. However, tests that assess the cellular response to the COVID-19 vaccines are not routinely available due to their relatively high costs and complicated testing processes.

## 5. Conclusions

To summarize the results of our research, we conclude the following:

1. COVID-19 vaccine hesitancy among parents of children after allo-HSCT poses a significant problem that complicates the prevention of SARS-CoV-2 infection in this high-risk group.
2. The COVID-19 mRNA BNT162b vaccine is safe and very well tolerated in 5–11-year-old children following allo-HSCT and in healthy children.
3. The 5–11-year-old patients who were more than 2 years removed from HSCT and who were not receiving immunosuppressive treatment presented excellent immunity after two doses of the mRNA BNT162b2 vaccine; thus, children in this group should be vaccinated according to the same schedule as the healthy population.
4. Some of the 5–11-year-old children after HSCT who fulfill the official criteria for the three-dose primary vaccination schedule might not need the third dose, and anti-SARS-CoV-2 IgG can be incorporated into decision making about the best vaccination schedule for such patients.

## Figures and Tables

**Table 1:** Characteristics of HSCT patients and the control group.

Parameter	HSCT Group	Control Group
Sex	male 28, female 6	male 17, female 18
Age	5–11 years (median 7)	5–11 years (median 7)
Diagnosis	severe aplastic anemia-10 acute lymphoblastic leukemia-6 juvenile myelomonycytic leukemia-4 acute myeloblastic leukemia-2 Wiskott–Aldrich syndrome-2 lymphoma-2 chronic myeloid leukemia-1 hemophagocytic syndrome-1 severe combined immunodeficiency-1 adrenoleukodystrophy-1 Diamond–Blackfan anemia-1 X-linked agammaglobulinemia-1 mucopolysaccharidosis-1	n/a
Time from allo-HSCT	5–120 months (median 36)	n/a
Symptoms of cGvHD at vaccination	present in 9 patients	n/a
Immunosupressive treatment at vaccination	9 patients receiving immunosuppressive treatment	n/a

**Table 2:** Anti-SARS-CoV-2 status before the vaccination and the immunologic answer to the vaccination.

Parameter	HSCT Group	Control Group	*p* Value
The number of patients with positive anti-SARS-CoV-2 IgG detected before vaccination	23/34	25/35	*p* = ns
The number of patients with anti-SARS-CoV-2 IgG detected after the second dose of the vaccine	34/34	35/35	*p* = ns
Anti-SARS-CoV-2 IgG concentration before one dose of the vaccine	0.1–1448 BAU/mL (median 75.4 BAU/mL)	0–1247 BAU/mL (median 81)	*p* = ns
Anti-SARS-CoV-2 IgG concentration after two doses of the vaccine	11.5–>5680 BAU/mL (median 3596)	1481–>5680 BAU/mL (median 3868)	*p* = ns
The number of patients who declared SARS-CoV-2 infection before the vaccination	8/34	10/35	*p* = ns

**Table 3:** Correlation results between lymphocyte subpopulations, IgG concentration, and pre- and post-vaccination anti-SARS-CoV-2 concentration in the HSCT group.

Value	SARS-CoV-2 Ab before Vaccination		SARS-CoV-2 Ab after 2nd Dose	
	Spearman r	*p* Value	Spearman r	*p* Value
T CD4+ lymphocytes (CD3 + 4+ cells/µL)	0.0092	0.96	0.1917	0.29
T CD8+ lymphocytes (CD3 + 8+ cells/µL)	−0.1274	0.48	−0.0035	0.98
NK cells (CD56+ cells/µL)	−0.0114	0.95	0.1387	0.44
B lymphocytes (CD19+ cells/µL)	0.3288	0.06	0.2236	0.21
IgG g/dL	−0.0836	0.64	0.1622	0.37

**Table 4:** The subgroup of HSCT patients who fulfilled the official criteria for the three-dose primary COVID-19 vaccine schedule.

The Reason Why the Patients Fulfilled the Criteria for the 3rd Dose of the Vaccine	**Less than 2 Years from HSCT**	**Still Receiving Immunosuppressive Treatment**	**Both Criteria Fulfilled**
	12/15	9/15	5/15
Anti-SARS-CoV-2 IgG concentration before the vaccination	1–1448 BAU/mL (3 patients negative, 12 patients >7 BAU/mL; median 66.8 BAU/mL)		
Anti-SARS-CoV-2 IgG concentration after the 2nd dose of the vaccine	11.5–>5680 BAU/mL (median 5574 BAU/mL)		
	**7–999 BAU/mL**	**1000–4999 BAU/mL**	**>5000 BAU/mL**
	7 patients	0 patients	8 patients
Anti-SARS-CoV-2 IgG after the 3rd dose of the vaccine	158–5680 BAU/mL (median 1240)	n/a	The 3rd vaccine dose was not given in the primary schedule due to excellent immunologic response to the 2nd dose

**Table 5:** Adverse events in the allo-HSCT and control groups (the white colour—HSCT group, the grey colour—CONTROL group).

Symptom	HSCT Patients Presenting the Symptom after the 1st Dose of the Vaccine (n = 34)	CONTROL Patients Presenting the Symptom after the 1st Dose of the Vaccine (n = 35)	HSCT Duration of the Symptom–Days after Vaccination–Median (Range)	CONTROL Duration of the Symptom–Days after Vaccination–Median (Range)	HSCT Patients Presenting the Symptom after the 2nd Dose of the Vaccine (n = 34)	CONTROL Patients Presenting the Symptom after the 2nd Dose of the Vaccine (n = 35)	HSCT Duration of the Symptom–Days after Vaccination–Median (Range)	CONTROL Duration of the Symptom–Days after Vaccination–Median (Range)	HSCT Patients in Whom the Symptom Was Present after both Doses (n = 34)	CONTROL Patients in Whom the Symptom Was Present After both Doses (n = 35)
Temp. 37.2–37.9 C	3 (8.8%)	1 (2.8%)	3 (1–4)	1 (1)	0	2 (5.7%)	-	3 (2–3)	1 (2.9%)	0
Temp. 38.0–38.4 C	0	0	-	-	0	1 (2.8%)	-	(3)	-	-
Fatigue	0	0	-	-	0	0	-	-	-	-
Headache	1	3 (8.5%)	(4)	2 (1–4)	2 (5.9%)	5 (14.2%)	(1–3)	2 (2–3)	1 (2.9%)	0
Chills	0	0	-	-	2 (5.9%)	3 (8.5%)	(1–3)	2 (1–3)	-	-
New or worsening muscle pain	2 (5.9%)	0	(2–4)	-	0	1 (2.8%)	-	(2)	-	-
New or worsening joint pain	0	0	-	-	0	0	-	-	-	-
Vomiting/nausea	0	0	-	-	1 (2.9%)	0	On 5th day	-	-	-
Pain at injection site	6 (17.6%)	7 (20.0%)	3 (1–4)	2 (1–4)	6 (17.6%)	13 (37.1%)	2 (1–2)	2 (1–5)	2 (5.9%)	4 (11.4%)
Swelling at the injection site	0	0	-	-	0	3 (8.5%)	-	5 (3–5)	-	-
Induration at the injection site	0	0	-	-	0	3 (8.5%)	-	5 (3–5)	-	-
Redness at the injection site	0	2 (5.7%)	-	(2–4)	1 (2.9%)	3 (8.5%)	(2)	3 (2–3)	0	0
Pruritus at the injection site	0	0	-	-	0	1 (2.8%)	-	(3)		-
Axillary lymphadenopathy	0	0	-	-	0	0	-	-		-

## Data Availability

The data presented in this study are available upon request from the corresponding author.
